# Ileocecal Fistula Caused by Multiple Foreign Magnetic Bodies Ingestion

**DOI:** 10.1155/2018/7291539

**Published:** 2018-01-23

**Authors:** Vittorio Cherchi, Gian Luigi Adani, Elda Righi, Umberto Baccarani, Giovanni Terrosu, Nicola Vernaccini, Vittorio Bresadola, Sergio Intini, Andrea Risaliti

**Affiliations:** Department of Medicine, University of Udine, P.le Kolbe, 33100 Udine, Italy

## Abstract

The incidence of accidental foreign body (FBs) ingestion is 100,000 cases/year in the US, with over than 80% of cases occurring in children below 5 years of age. Although a single FB may pass spontaneously and uneventfully through the digestive tract, the ingestion of multiple magnetics can cause serious morbidity due to proximate attraction through the intestinal wall. Morbidity and mortality depend on a prompt and correct diagnosis which is often difficult and delayed due to the patient's age and because the accidental ingestion may go unnoticed. We report our experience in the treatment of an 11-year-old child who presented to the emergency department with increasing abdominal pain, vomiting, diarrhea, and fever. Surgery evidenced an ileocecal fistula secondary to multiple magnetic FB ingestion with attraction by both sides of the intestinal wall. A 5-centimeter ileal resection was performed, and the cecal fistula was closed with a longitudinal manual suture. The child was discharged at postoperative day 8. After one year, the patient's clinical condition was good.

## 1. Introduction

The ingestion of foreign bodies (FBs) is common in children, especially between 6 months and 3 years of age [1]. Most pediatric ingestions are accidental (98% of cases) [2]. Coins and battery represents the most frequently ingested objects, followed by toys, magnetics, sharp objects, caustic liquids, batteries, and food [3]. During the last decade, powerful and small rare-earth magnets have been manufactured and incorporated into toys, thus increasing the risk of ingestion of dangerous material [4]. Most of FBs pass through the gastrointestinal tract uneventfully; however, in rare cases, their shape and size can cause complications such as obstruction, ischemia, perforation, or fistula [5]. Endoscopic intervention is required in 10–20% of patients, while only 1% of patients need urgent surgical approach [6]. Although ingestion of a single magnetic FB may, in most cases, be managed as a simple FB ingestion, multiple magnetic FBs are associated with an elevated risk of complications [7]. Two or more magnets separated along their course in the gastrointestinal tract may attract the bowel walls, causing pressure necrosis and subsequent small bowel obstruction, volvulus, fistula, or perforation [8].

## 2. Case Report

An 11-year-old child was admitted to the emergency department with a 3-day history of abdominal pain. Clinical examination showed abdominal tenderness, normal peristalsis, and no evidence of peritoneal irritation. Laboratory exams evidenced an increased white blood cell count (WBC 13.810/mmc with neutrophilia 91%), while C-reactive protein was within the normal range (5 mg/L). Abdominal ultrasound, limited by the abdominal meteorism and intestinal content, only showed the presence of fluid in the pouch of Douglas. During the observation, the child presented progressively worsening conditions with vomiting, diarrhea, and fever (>38°C). The abdominal pain worsened and was associated with peritoneal irritation in right iliac fossa with positive McBurney's sign.

**Figure 1:**
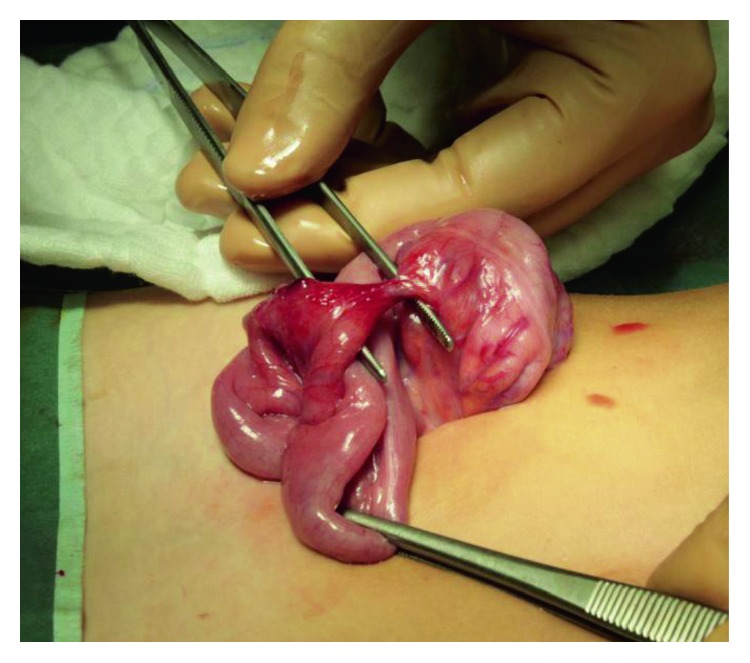
Ileocecal fistula.

The child underwent urgent examination for suspected acute appendicitis complicated by peritonitis. A McBurney's incision was performed showing normal appendix but the presence of an ileocecal fistula of 2 cm. The fistula was determined by bowel wall necrosis due to proximate attraction through the intestinal wall (Figure 1). We performed a resection of 5 cm of ileum involving the fistula with ileo-ileal termino-terminal anastomosis using a double running suture to restore intestinal continuity. The cecal fistula was closed with a longitudinal single-manual running suture.

**Figure 2:**
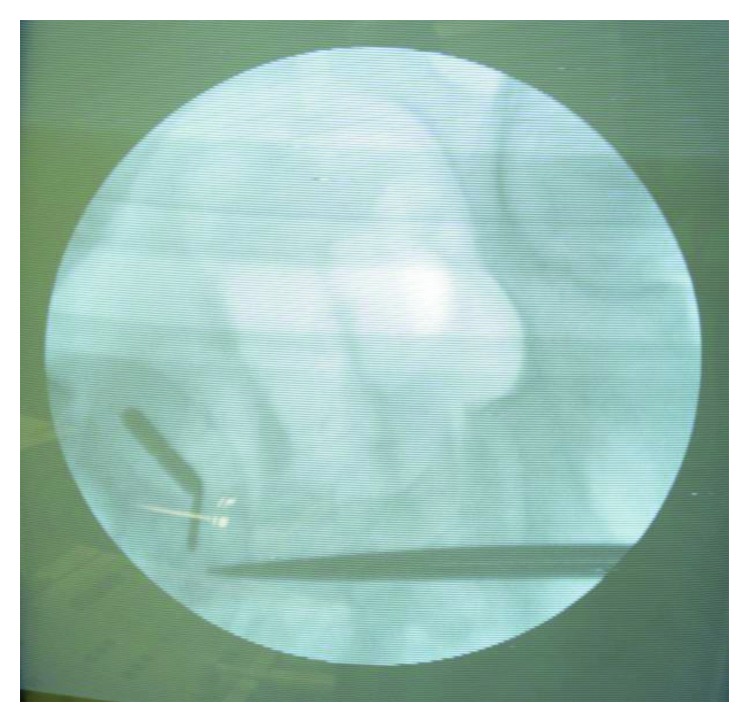
Intraoperative X-ray evidenced 2 magnets in the ileum.

**Figure 3:**
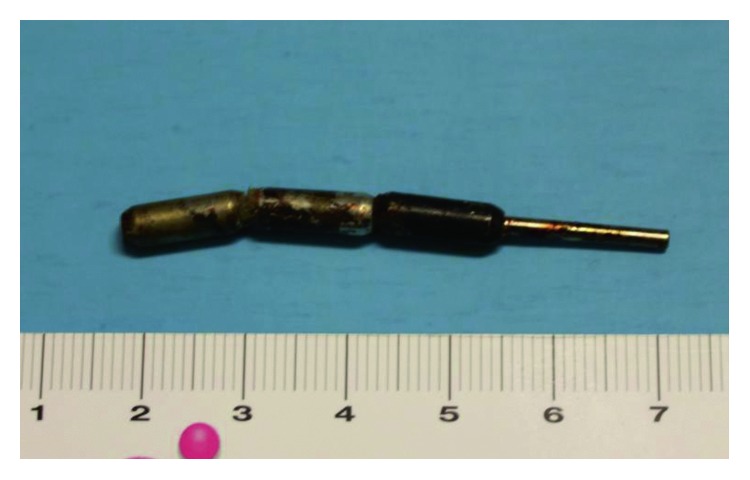
Magnets removed by the bowel.

An intraoperative X-ray evidenced other 2 magnets in the ileum, thirty and forty centimeters from the ileocecal valve, respectively, which were promptly removed performing two little enterotomy (Figures 2 and 3).

The patient had an uneventful postoperative course. A few days after the operation, the patient admitted that he ingested two pieces of metal almost two months before.

The child resumed bowel function 72 hours later, and he was discharged from hospital after postoperative day 8. The patient's clinical condition was good at one-year follow-up.

## 3. Discussion

Accidental foreign body ingestion is a widespread problem in infants and childhood [1]. The symptoms depend on the type, size, and site of the foreign body, although more than 80% of cases result in a spontaneous passage through the gastrointestinal tract without complications [6].

Usually, the ingestion of a single small magnetic foreign body does not cause damage to the gastrointestinal tract [3]. On the contrary, the ingestion of multiple magnetic bodies could be dangerous and requires special consideration for possible complications [9]. Some reports highlight how the ingestion of 2 magnets at the same time can be dangerous because in this situation the magnets can stick together in the stomach [10]. When the ingestion is asynchronous, the magnets can be attracted mutually, anchoring bowel walls and exerting pressure, with necrosis of bowel walls, as evidenced in our case. The consequences can lead to perforation, intestinal obstruction, volvulus, and fistula [11].

In a report, delayed diagnosis (longer than two days from ingestion) and treatment were related with a worse outcome [5]. The symptoms usually appear between 1 and 7 days after ingestion [12], but some authors reported delayed diagnosis of ingested foreign body in a child with intermittent abdominal pain of 6 months [13]. Some reports showed that the small bowel, close to ileocecal valve, represents the area of the intestinal tract more involved [14, 15].

In the suspicion of foreign body ingestion, an X-ray should be always performed, even in older children [6]. In our case, we initially did not perform the abdominal X-ray because the child denied having ingested any FB, and the symptoms were compatible with complicated acute appendicitis. Only after the operation, the patient admitted the ingestion of the first two pieces of metal, almost two months before.

There is no consensus for the management of magnetic FB ingestion in children. In case of ingestion of multiple magnetic FBs, their removal is recommended before symptoms occur, either by endoscopy or by laparotomy [16]. For ingestion of single magnetic FB, recommendations include close observation in asymptomatic patients, repeated abdominal radiographs within a few hours to assure advancement of the FB through the gastrointestinal tract, and immediate endoscopic or surgical removal in case of perforation or obstruction [15].

## 4. Conclusion

The ingestion of a foreign body is a common occurrence, which can result in intestinal obstruction, perforation, or fistula. Although ingestion of a single magnetic FB may, in most cases, be managed as a simple FB ingestion, the ingestion of multiple magnetic FBs is associated with an elevated risk of complications and requires aggressive management. Early endoscopic or surgical approach is important to reduce morbidity and mortality. In children with a noncharacterized abdominal pain, the foreign body ingestion should always be suspected. FB ingestion must not be excluded also in case of older children, even in presence of denial and may represent a previously not known neuropsychiatric problem. Pediatricians and surgeons should take a vigilant approach to this problem, especially in cases with an unclear clinical or radiological diagnosis of acute and recurrent abdominal pain.

## References

[1] Litovitz T. L., Klein-Schwartz W., White S. (2001). 2000 annual report of the American Association of Poison Control Centers Toxic Exposure Surveillance System. *American Journal of Emergency Medicine*.

[2] Suk-Koo L., Nam-seon B., Hyun-Hahk K. (1996). Mischievous magnets, unexpected health hazard in children. *Journal of Pediatric Surgery*.

[3] Erbes J., Babbit D. P. (1965). Foreign bodies in the alimentary tract of infants and children. *Applied Therapeutics*.

[4] Hachimi-Idrissi S., Corne S., Vandenplas Y. (1998). Management of ingested foreign bodies in childhood: our experience and review of the literature. *European Journal of Emergency Medicine*.

[5] Cho J., Sung K., Lee D. (2017). Magnetic foreign body ingestion in pediatric patients: report of three cases. *BMC Surgery*.

[6] Lai A. T., Chow T. L., Lee D. T., Kwok S. P. Y. (2003). Risk factors predicting the development of complications after foreign body ingestion. *British Journal of Surgery*.

[7] Kay M., Wyllie R. (2002). Techniques of foreign body removal in infants and children. *Techniques in Gastrointestinal Endoscopy*.

[8] Chang W. J., Chiu W. Y. (2017). Gastric foreign body: a comb. *Clinical Case Reports*.

[9] Ilce Z., Samsum H., Mammadov E., Celayir S. (2007). Intestinal volvulus and perforation caused by multiple magnet ingestion: report of a case. *Surgery Today*.

[10] Dutta S., Barzin A. (2008). Multiple magnet ingestion as source of severe gastrointestinal complications requiring surgical intervention. *Archives of Pediatrics & Adolescent Medicine*.

[11] Anselmi E., San Roman C. G., Barrios Fontoba J. E. (2007). Intestinal perforation caused by magnetic toys. *Journal of Pediatric Surgery*.

[12] Chung J. H., Kim J. S., Song Y. T. (2003). Small bowel complication caused by magnetic foreign body ingestion of children: two case reports. *Journal of Pediatric Surgery*.

[13] Butterworth J., Feltis B. (2007). Toy magnet ingestion in children: revising the algorithm. *Journal of Pediatric Surgery*.

[14] Fenton S. J., Torgenson M., Holsti M. (2007). Magnetic attraction leading to a small bowel obstruction in a child. *Pediatric Surgery International*.

[15] Tavarez M. M., Saladino R. A., Gaines B. A., Manole M. D. (2013). Prevalence, clinical features and management of pediatric magnetic foreign body ingestions. *Journal of Emergency Medicine*.

[16] Nui A., Hirama T., Katsuramaki T. (2005). An intestinal volvulus caused by multiple magnet ingestion: an unexpected risk in children. *Journal of Pediatric Surgery*.

